# Perception of biological motion from size-invariant body representations

**DOI:** 10.3389/fnint.2015.00024

**Published:** 2015-03-24

**Authors:** Markus Lappe, Karin Wittinghofer, Marc H. E. de Lussanet

**Affiliations:** Institute for Psychology, University of MuensterMuenster, Germany

**Keywords:** action recognition, biological motion perception, template matching, size invariance, point-light animations

## Abstract

The visual recognition of action is one of the socially most important and computationally demanding capacities of the human visual system. It combines visual shape recognition with complex non-rigid motion perception. Action presented as a point-light animation is a striking visual experience for anyone who sees it for the first time. Information about the shape and posture of the human body is sparse in point-light animations, but it is essential for action recognition. In the posturo-temporal filter model of biological motion perception posture information is picked up by visual neurons tuned to the form of the human body before body motion is calculated. We tested whether point-light stimuli are processed through posture recognition of the human body form by using a typical feature of form recognition, namely size invariance. We constructed a point-light stimulus that can only be perceived through a size-invariant mechanism. This stimulus changes rapidly in size from one image to the next. It thus disrupts continuity of early visuo-spatial properties but maintains continuity of the body posture representation. Despite this massive manipulation at the visuo-spatial level, size-changing point-light figures are spontaneously recognized by naive observers, and support discrimination of human body motion.

## 1. Introduction

The destinction between form perception and motion perception has been of fundamental importance for our understanding of the structure of the human visual system. Biological motion perception, the perception of the action of other humans or animals, brings form and motion together. The reason is that the movements of the body are characterized by its shape and by the constraints that the joints put on the motion of the limbs. Biological motion is often investigated using point-light figures. In point-light figures the body itself is invisible. Instead, a small number of points at the major joints provide information about the position and motion of these joints. Such point-light figures contain little information about the form of the body, for example they do not show the edges of the limbs, but each point presents the correct motion signal from a particular joint. Therefore, the integration of the light points for the recognition of a human figure was originally believed to rely on an analysis of the motion patterns of the individual points with respect to each other (Johansson, 1973; Cutting et al., 1988). However, several findings with different methodological approaches have cast doubt on this view. First, studies in patients with severe deficits in motion perception showed that biological motion perception may be spared even when general motion perception is impaired (Vaina et al., 1990; McLeod et al., 1996; Schenk and Zihl, 1997; Vaina et al., 2002; Huberle et al., 2009). Second, a variation of the point-light stimulus in which local motion of the points is rendered uninformative was shown to provide enough information for biological motion perception (Beintema and Lappe, 2002; McKay et al., 2009; Lu, 2010; Thirkettle et al., 2010; Theusner et al., 2011). Moreover, transcranial magnetic stimulation to inactivate area V5/MT did not interfere with biological motion perception (Grossman et al., 2005). These results showed that there must be a route to biological motion that does not use the local motion of the individual points but instead uses form information from the global configuration of the points (Beintema and Lappe, 2002). Since then, there has been an extensive discussion about the respective contributions of form and motion cues to different tasks of biological motion perception which made clear that there are multiple routes to biological motion. For example, local motion trajectories of individual points, such as the feet, carry kinematic and dynamic information that support a percept of animacy (Chang and Troje, 2008) and allow the discrimination of facing direction (Troje and Westhoff, 2006). On the other hand, facing direction can also be retrieved from static global form information (Lange and Lappe, 2007; Reid et al., 2009). Moreover, local motion has been shown to interact with form cues to biological motion perception (Thurman and Lu, 2013).

The form pathway to biological motion perception involves the analysis of body posture and its change over time (Beintema and Lappe, 2002; Giese and Poggio, 2003; Lange et al., 2006). In this view, each static image of a walker signals a particular posture of the body. A series of such images signals a particular body movement. The perception of biological motion can then be accomplished via a sequence analysis of the posture information. Such a procedure needs mechanisms to identify body form and posture. Indeed, biological motion stimuli activate brain areas that also respond to the view of human bodies (Perrett et al., 1990; Downing et al., 2001; Michels et al., 2005; Peelen and Downing, 2005; Jastorff and Orban, 2009; Vangeneugden et al., 2011; Jastorff et al., 2012). Moreover, psychophysical experiments have shown that biological motion recognition is susceptive to interference from object recognition mechanisms specifically when the interfering stimuli are images of the human body (Wittinghofer et al., 2010, 2012). One possibility to identify body form and posture is via established hierarchical object recognition processes in the ventral stream of visual cortex, starting from simple cell edge detectors and working up to intermediate and full-body stages (Giese and Poggio, 2003). For point-light stimuli, however, these processes are ineffective because these stimuli lack edge information (Giese and Poggio, 2003). Moreover, patients with deficits in hierarchical form processing for object recognition can still perceive biological motion (Vaina et al., 2002; Gilaie-Dotan et al., 2011, 2015; Huberle et al., 2012). Another possibility is that the shape information in point-light figures is picked up from a direct matching of the point-light positions to templates of the human body (Lange and Lappe, 2006). This approach bears some resemblance to configural processing in face perception as individuals suffering from congenital prosopagnosia also showed specific impairments in biological motion perception (Lange et al., 2009).

**Figure 1:**
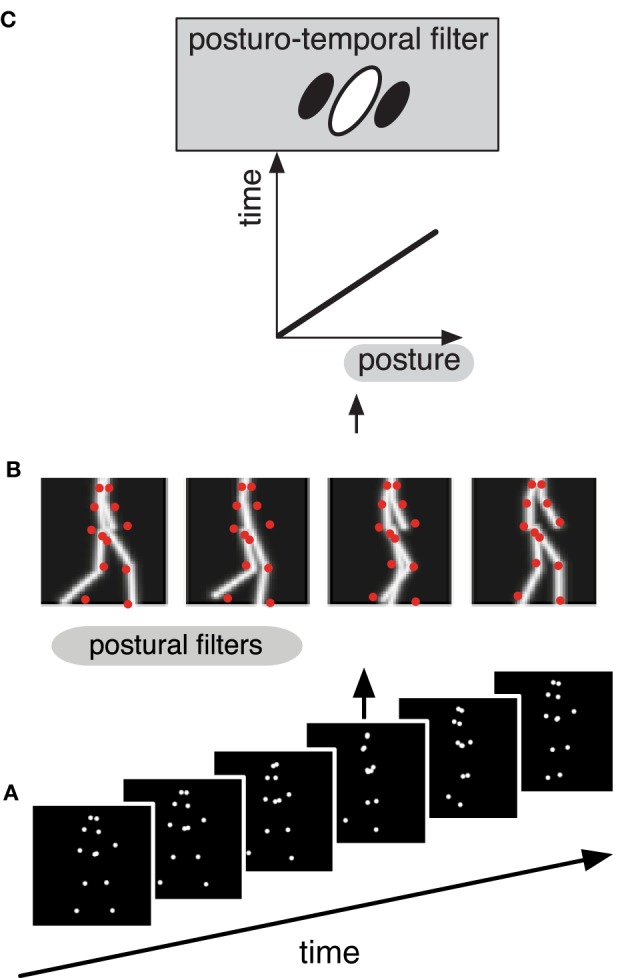
**Schematic depiction of the hierarchical processing (A–C) in the different stages of the postural-temporal filter model of biological motion perception (Theusner et al., 2014). (A)** The stimulus consists of a sequence of point-light images. **(B)** Each single frame from the sequence (red dots) is matched against a representation of postures (gray lines) by a set of postural filters. **(C)** The signal of these postural filters is then fed into the posturo-temporal filter representation of an action (i.e., walking), which represents the temporal order of the templates of the posture space.

The posturo-temporal filter model of biological motion recognition (Theusner et al., 2014) assumes a cortical representation of the postures that the body takes during an action, for example during walking (Figure 1). In the first step, each static frame of the animation of a point light walker (Figure 1A) is matched in parallel against all the templates in this posture space (Figure 1B). This creates a distribution of activity in posture space over time. This stage is sufficient to perform shape based discriminations of point-light figures such as the discrimination of facing direction or the presence of a human figure, but not its motion. In analogy to the standard motion energy model the next processing step may be called posturo-temporal filtering and involves the application of Gabor-type filters along the temporal and the postural dimensions to estimate the change of posture over time, i.e., the motion of the body (Figure 1C). Detailed analysis of the properties of this model revealed considerable similarity to the properties of body and action selective neurons in the temporal cortex of the macaque monkey (Theusner et al., 2014).

A characteristic feature of this model is that it begins with an analysis of the static form of the human body. Since this first stage of body form analysis is distinct from the subsequent body motion analysis one might expect that it shows typical properties of form analysis. One such property is size invariance. Indeed, we easily recognize the human form in different retinal sizes, and representations of biological motion in the STS show a certain amount of size invariance (Ashbridge et al., 2000; Grossman et al., 2010). However, although size invariance is presently not implemented in the model, potential implementations discussed in Theusner et al. (2014) suggest a prediction that goes beyond just size invariance of point-light walkers: If biological motion perception is derived from a representation of body postures, and if this representation were by itself size invariant, then biological motion perception should also be *invariant to size changes for each body posture in a sequence*. To put it differently, biological motion recognition from a sequence of body postures images should work even when each image in the sequence has a different size.

We have created point-light animations in which the size of a walking human figure changes from every animation frame to the next. We show that human observers spontaneously recognize these as human figures, and that they can determine facing direction and walking movement from these animations. We conclude that the mechanism of biological motion perception include a representation of posture that is size invariant and precedes motion recognition.

## 2. Materials and methods

We report two experiments. The first experiment tested spontaneous recognition of point-light walkers that change randomly in size from frame to frame. The second experiment tested the discrimination of facing and walking direction of these stimuli.

### 2.1. Experiment 1: spontaneous recognition

In the spontaneous recognition experiment we tested whether naive subjects who were not familiar with point-light stimuli spontaneously recognized the size-changing stimulus as a human actor.

#### 2.1.1. Stimuli

**Figure 2:**
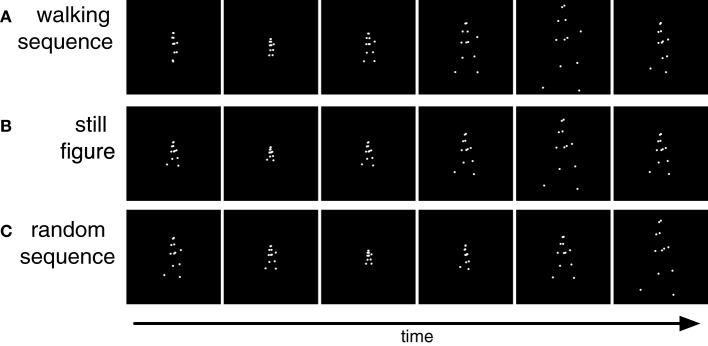
**Stimuli used in Experiment 1. (A)** Sequence of point light walking in which each frame is scaled differently from the preceding frame. **(B)** Sequence of differently scaled figures of a single static posture. **(C)** Sequence of differently scaled frames of the postures of the walking cycle in random temporal order.

Stimuli (Figure 2) were taken from a set of motion tracking data of a walking humans that we used in earlier studies (de Lussanet et al., 2008; Kuhlmann et al., 2009; Lange et al., 2009; Michels et al., 2009). They showed the walker in side view and were presented as a frame-by-frame animation on a MacBook (1280 × 800 pixels, 60 Hz) viewed from 90 cm distance to the screen. Horizontal translation was subtracted from the recorded walking, so that it looked like walking on a treadmill. Each stimulus frame showed 12 white (11 cd/m^2^) points (0.2° diameter) on a black (0.04 cd/m^2^) background representing the positions of shoulders, hips, elbows, wrists, knees, and ankles of a walker for 100 ms. The subsequent frame then showed the next (for forward walking) or previous (for backward walking) posture from the walking cycle with the points at the same positions on the body. We chose a frame duration of 100 ms to avoid interference from visible persistence at short frame durations. The stimulus showed a singe gait cycle (two steps, 1.6 s) in a looped presentation that lasted for 60 s. It was originally recorded at 86 Hz and interpolated to the frame rate of the experiment.

The size of the walker varied between 1.6° and 7.3° in different frames. The size of the first frame was chosen randomly within this range. Walker size was then randomly up- or down-scaled from frame to frame by a factor of 1.75 (Figure 2A). If the scaling would result in walker sizes outside the maximum or minimum size the scaling was reversed to keep the stimulus within the prescribed range. Besides this walking sequence we constructed two further stimuli. The *still figure* stimulus (Figure 2B) showed only one characteristic static body posture (arms and legs extended) which changed in size from frame to frame but did not walk. In the *random sequence* stimulus (Figure 2C) all body postures from the walking sequence were presented with changing size but in randomized temporal order.

#### 2.1.2. Participants

A total of 151 observers (60 male, average age 25.2 ± 5.42 years) participated in the experiment. All had normal or corrected-to-normal vision. Experiments adhered to the required standards set by the ethical committee of the department. Participants gave informed consent, were allowed to withdraw from the study and received full de-briefing if they wished. Data was anonymized.

#### 2.1.3. Procedure

Each participant saw only one stimulus either the walking sequence (86 participants) or the still figure (44 participants), or the random sequence (21 participants). At first the experimenter explained to the subject that in the following he or she would see a short visual presentation. Subjects were then asked to watch the stimulus and to write down a description of what they see. At the end of the experiment, subjects were asked to note if they had seen a similar presentation before and if this was the case, in which context. Subjects entered data analysis only if they had not seen a point-light stimulus before.

The written descriptions were evaluated by two experimenters. Descriptions that referred to a human figure or human actions were rated as successful recognition. Examples for successful recognition include: a jogger, a walking human seen near or far, a dancer, etc. A description was counted as successful only if both raters independently rated it as successful recognition. Examples of descriptions that were classified as not recognized included: the Eiffel tower in different sizes, an illustration of sound, fireworks, or white dots on black background which alternate between convergence and divergence.

If the experimenter had the impression, that the participant had not recognized the stimulus as a walking human she presented a classic walker of uniform size as second stimulus and again asked the participant to provide a written description. This second presentation served as a control for the general ability of recognizing a walker from a point light display. The classic walker was presented in the middle of the screen. The walker size on screen was 2.5 cm (1.6° viewing angle). The size and position remained the same over all frames. Only one of the subjects did not recognize this stimulus as a walking human. This result ensures that the subjects which had not recognized the size-changing walker as a human figure were in general able to recognize a human walker from a point-light display.

### 2.2. Experiment 2: discrimination tasks

In the discrimination experiments, we investigated whether observers could discriminate facing and walking direction of the size-changing stimulus.

#### 2.2.1. Stimuli

Stimuli were presented on an Iiyama Vision Master 505 computer monitor (CRT, 1024 × 768 pixels, 100 Hz) that was viewed from about 57 cm distance to the screen. The stimulus was constructed in the same way as in the spontaneous recognition experiments, but with a luminance of 30 cd m^−2^ for the dots and 0.1 cd m^−2^ for the background. Also, stimulus size (between 1.9° and 17.5°), frame duration (60 ms) and frame-to-frame scaling were slightly different. The scaling factor was chosen randomly between 1.7 and 1.8 to avoid the possibility that the exact same walker size was presented twice in a single trial.

#### 2.2.2. Participants

Twelve female students of the University of Münster between 19 and 30 years old (average 24 years) participated for course credit. All participants had normal or corrected-to-normal vision.

#### 2.2.3. Procedure

The experiment contained two tasks run in separate sessions. In the facing task, stimuli showed either leftward or rightward facing walkers in forward walking sequence. Participants were asked to report the facing direction. In the walking task, stimuli showed rightward facing walkers that walked either forward or backward. Participants were asked to report whether the stimulus walked forward or backward.

Stimuli were shown in blocks of 150 trials. Each trial presented a single stimulus for 1 s. After each stimulus presentation the subject gave a manual responses with her right hand by pressing the arrow keys of the keyboard connected to the operating computer (Apple Mac-Book Pro). Before the data collection started, the subject familiarized herself with the tasks in two blocks of practice trials (one for each task) with a walker of a fixed size of 4.8°. Thereafter she performed the two sessions. Session order was counterbalanced over subjects.

To test if the subjects performance was significantly higher than chance level, we calculated *t*-tests in which the means of the groups were compared to chance level (criterium = 0.5). Furthermore, we calculated paired sample *t*-tests to compare the performance between the tasks.

## 3. Results

### 3.1. Experiment 1: spontaneous recognition

**Figure 3:**
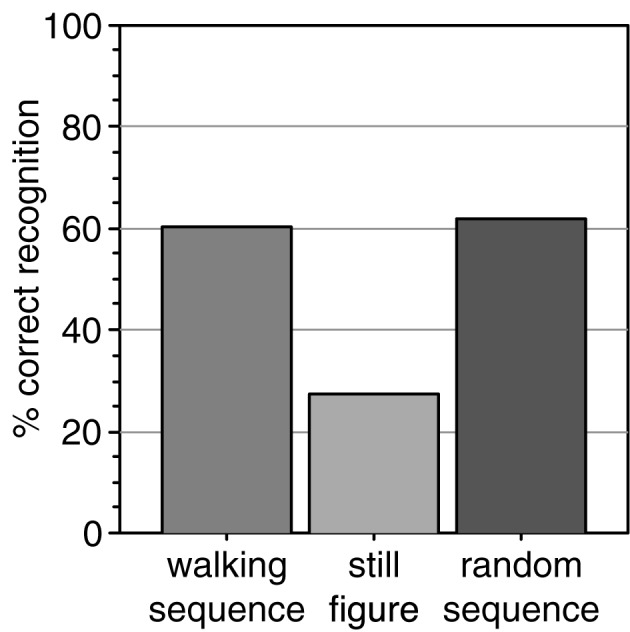
**Percentage of observers that spontaneously reported to perceive a human actor from the size-changing walking sequence**. Comparison data was collected for a sequence of size-changing still images of a single posture and of a sequence of random postures.

We tested whether point-light walkers that randomly change in size from frame to frame would spontaneously induce the percept of biological motion. We presented such stimuli to 100 naive observers and asked them to write down a description of what they saw. In the instruction, no reference was made to biological motion, a human figure, or any action. More than half of the subjects (60%) spontaneously reported to see a human figure, despite the random changes in size (Figure 3).

To test whether the walking motion contributed to the recognition we tested two further stimuli. The still frame stimulus showed a characteristic single posture from the walking cycle (arms and legs extended) which changed in size from frame to frame but did not perform the walking motion. This stimulus was shown to a new group of naive observers. Only 27% recognized it as a human figure, significantly less than in the walking condition [Figure 3, chi square test, chi(1) = 12.8; *p* < 0.001]. Thus, a set of different postures led to better recognition than presentation of a single posture.

The third stimulus tested whether an orderly walking sequence was necessary for the recognition. Since the second stimulus used a single posture, it may be that this single posture was insufficient to induce the percept. Therefore, we tested whether accumulating otherwise randomly presented frames results in the perception of a human figure. To investigate this, we constructed an animation in which all postures of the walking cycle were used with randomly changing size, but not in the correct temporal order of the walking movement but rather in a random sequence. Even this stimulus was described as a human figure by a majority (62%) of a new set of naive observers (Figure 3), significantly more than the static stimulus [chi(1) = 7.2; *p* = 0.007] and not different from the normal walking stimulus [chi(1) = 0.01; *p* = 0.90].

These results show that a rapid series of point-light images in different postures suffices to induce the percept of a human figure in naive observers. However, it is not necessary that the posture images are arranged in a continuous walking movement. Consistent with the first stage of the model, this suggests that the perception of a human figure is supported by the static posture information in each image. Moreover, a set of different postures yields better recognition than repetition of the same posture. From the view point of the model this could indicate that stimulation of a larger part of the posture representation leads to better recognition or that some form of probability summation from different static frames takes place.

To summarize, the results of Experiment 1 show that body posture information from different sizes and postures can be accumulated to induce the percept of a human figure. Experiment 2 tests whether the temporal arrangement of walking motion can also be perceived from the size changing stimuli, i.e., whether the size-changing presentation truly allows the perception of body motion.

### 3.2. Experiment 2: discrimination of facing and walking direction

Experiment 2 tested two discrimination tasks with the size-changing walker stimuli. In the facing discrimination, stimuli showed either leftward or rightward facing size-changing walkers in forward walking sequence. Participants had to indicate the facing direction. In the walking direction experiment, stimuli showed size-changing walkers that walked forward or backward. Participants had to indicate the walking direction. Walker size varied between 1.9° and 17.5° from frame to frame. From frame to frame (60 ms) the stimulus was randomly up- or down-scaled by a factor between 1.7 and 1.8.

**Figure 4:**
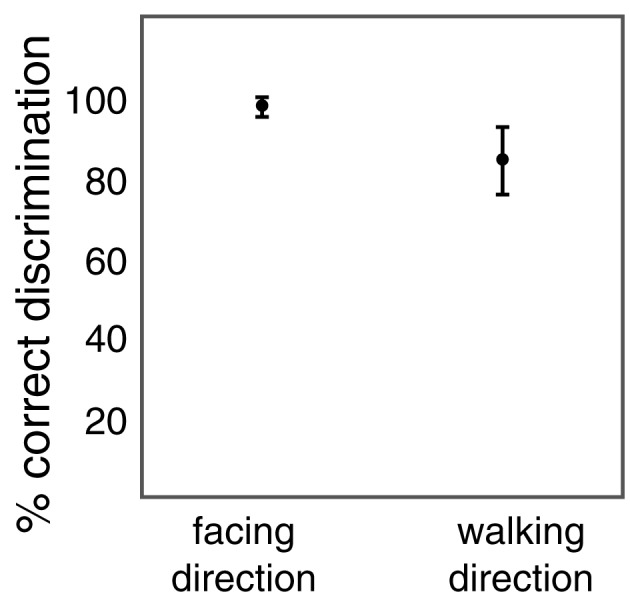
**Percentage of correct discrimination of facing direction (left vs. right) and of walking direction (forward vs. backward) of the size-changing point-light walker**. Error bars show standard deviations.

Figure 4 shows the results from the two tasks. Participants were on average 97% correct in the facing discrimination and 84% correct in the walking discrimination. Both values are significantly above chance level (*t*-tests: *t*_(11)_ = 66.924; *p* < 0.001 for facing; *t*_(11)_ = 13.948; *p* < 0.001 for walking). Thus, both facing and walking direction discrimination were possible despite the rapid changes in size of the posture frames. The difference in performance between facing and walking discrimination (*t*-test: *t*_(11)_ = 6.145; *p* < 0.001) is consistent with the previous observation that walking discrimination is more difficult than facing discrimination and requires an additional processing stage (Beintema et al., 2006; Lange and Lappe, 2006; Wittinghofer et al., 2010). However, the above chance performance in walking discrimination shows that observers not only recognized the human form from these animations but also perceived the motion of the body.

## 4. Discussion

Our results show that several aspects of biological motion can be perceived from point-light animations that change in size from frame to frame. Naive subjects can recognize them as human actors. A stimulus set of several different postures is better recognized than a single posture, even if these postures are not in the correct temporal order of the walking cycle. Yet, temporal order of these stimuli can be perceived since otherwise subjects would not have been able to reach above chance performance in the walking direction task. These results provide important data for a future implementation of size-specificity in the posturo-temporal filter model of biological motion perception. They suggests that size invariance either acts on each individual posture before they are bound together to the percept of biological motion, or that the posturo-temporal filters receive input from posture templates of different sizes.

The finding that the human figure is recognized in either the correct or a random sequence of postures is consistent with the template model of biological motion recognition (Lange and Lappe, 2006; Theusner et al., 2014) because in this model each frame of the point-light stimulus is initially analyzed separately and independently of its position in the sequence. Perception of figural information, for example facing direction, is supported by this first stage of the model, and the stimulation of a set of posture templates in random order suffices to determine a match with the characteristics of the human body (Lange and Lappe, 2007). Perception of body motion in the walking direction task relies on temporal sequence analysis.

The large size changes from frame to frame destroy motion correspondences of individual points between frames, thus rendering local motion useless. This is a similar effect as the lifetime reduction used by Beintema and Lappe (2002) and re-iterates their conclusion that local motion signals are not necessary for biological motion perception. However, the current data makes two additional points. First, even the normal point light walker with points on the joints carries sufficient form information to allow spontaneous recognition by naive observers, as it does for experienced observers (Cutting et al., 1988; Reid et al., 2009). Second, the recognition of a human figure, of its facing direction, and of its walking direction combines information from different stimulus sizes.

Biological motion is such a strong percept that normally recognition and discrimination rates are close to perfect. For example, Beintema and Lappe (2002) found that 84% of naive observers spontaneously recognized a normal point-light walker that did not change in size. The discrimination of walking direction for normal point-light walkers is also close to 100% correct (e.g., Kuhlmann et al., 2009). For the size-changing walkers, recognition by naive subjects and discrimination performances were below those values, showing that biological motion perception is impaired for these stimuli. Some impairment is likely to be expected given the limits of size invariance in the neural representations in the object recognition pathways of the brain. Although body-form selective neurons in the STS show considerable size invariance, not all neurons do, and the response rates of those neurons that show invariance still vary with size (Ashbridge et al., 2000).

In relating our results to the neuroscience of biological motion perception we have to consider possible neural structures for the two stages of the postural-temporal filter model. Theusner et al. (2014) suggested that posture-selective neurons (implementing the postural filters in Figure 1B) and body motion-selective neurons (implementing the posturo-temporal filters in Figure 1C) correspond to “static-action” and “action” neurons described by Vangeneugden et al. (2011) in the monkey. Both types of neurons were found in the upper bank of the STS [consistent with other reports of both static and action selectivity in upper bank of the STS (Perrett et al., 1990; Jellema and Perrett, 2003; Singer and Sheinberg, 2010)]. Static-action neurons were also prominent in the lower bank. Jastorff et al. have suggested that the lower bank of monkey STS corresponds in humans to body selective areas in the posterior infero-temporal sulcus (including EBA) and the fusiform gyrus (including FBA) (Jastorff and Orban, 2009; Jastorff et al., 2012). Thus, either one of these areas could correspond to the posture-selective stage, or, alternatively, the posture-selective stage could involve posture-selective neurons directly in the pSTS intermixed with the body motion-selective neurons. Some degree of size invariance to body stimuli has been described in all three areas (Ashbridge et al., 2000; Aleong and Paus, 2010). Whether EBA contributes to action recognition is currently debated. It contains representations of body form and body motion (Jastorff and Orban, 2009), but these can be segregated in multi-voxel analysis (Thompson and Baccus, 2012; Vangeneugden et al., 2014) and TMS inactivation over EBA does not interfere with body motion perception (Vangeneugden et al., 2014). Recent patient studies have shown that lesions in EBA do not impair biological motion perception as much as lesions in pSTS (Gilaie-Dotan et al., 2015). The same was found for lesions in other ventral stream areas, including FBA (Gilaie-Dotan et al., 2015). This could be explained if multiple routes to biological motion perception exist, in which case lesions in any one particular pathway do not abolish perception. However, it is also possible that size-invariant biological motion recognition is achieved from template-matching directly in the pSTS as both posture-selective neurons and body motion-selective neurons have been found there.

### Conflict of interest statement

The authors declare that the research was conducted in the absence of any commercial or financial relationships that could be construed as a potential conflict of interest.
